# Crystal structure and functional analysis of mycobacterial erythromycin resistance methyltransferase Erm38 reveals its RNA-binding site

**DOI:** 10.1016/j.jbc.2022.101571

**Published:** 2022-01-08

**Authors:** Boon Chong Goh, Xinyu Xiang, Julien Lescar, Peter C. Dedon

**Affiliations:** 1 Antimicrobial Resistance Interdisciplinary Research Group, Singapore-MIT Alliance for Research and Technology Centre, Singapore, Singapore; 2 NTU Institute of Structural Biology, Experimental Medicine Building (EMB), Nanyang Technological University, Singapore, Singapore; 3 School of Biological Sciences, Nanyang Technological University, Singapore, Singapore; 4 Department of Biological Engineering, Massachusetts Institute of Technology, Cambridge, Massachusetts, USA

**Keywords:** antibiotic resistance, RNA-binding protein, RNA methyltransferase, X-ray crystallography, molecular dynamics, mycobacteria, erythromycin resistance methyltransferase, AMR, antimicrobial resistance, Erms, Erythromycin resistance methyltransferases, IMAC, immobilized metal affinity chromatography, MLS, macrolides, lincosamides, and streptogramins, PBST, phosphate-buffered saline with Tween, VMD, visual molecular dynamics

## Abstract

Erythromycin resistance methyltransferases (Erms) confer resistance to macrolide, lincosamide, and streptogramin antibiotics in Gram-positive bacteria and mycobacteria. Although structural information for ErmAM, ErmC, and ErmE exists from Gram-positive bacteria, little is known about the Erms in mycobacteria, as there are limited biochemical data and no structures available. Here, we present crystal structures of Erm38 from *Mycobacterium smegmatis* in apoprotein and cofactor-bound forms. Based on structural analysis and mutagenesis, we identified several catalytically critical, positively charged residues at a putative RNA-binding site. We found that mutation of any of these sites is sufficient to abolish methylation activity, whereas the corresponding RNA-binding affinity of Erm38 remains unchanged. The methylation reaction thus appears to require a precise ensemble of amino acids to accurately position the RNA substrate, such that the target nucleotide can be methylated. In addition, we computationally constructed a model of Erm38 in complex with a 32-mer RNA substrate. This model shows the RNA substrate stably bound to Erm38 by a patch of positively charged residues. Furthermore, a π-π stacking interaction between a key aromatic residue of Erm38 and a target adenine of the RNA substrate forms a critical interaction needed for methylation. Taken together, these data provide valuable insights into Erm–RNA interactions, which will aid subsequent structure-based drug design efforts.

The emergence of antimicrobial resistance (AMR) in virtually every clinically important bacterial pathogen represents a true crisis with major societal and economic impact (1) (https://amr-review.org/sites/default/files/AMR%20Review%20Paper%20-%20Tackling%20a%20crisis%20for%20the%20health%20and%20wealth%20of%20nations_1.pdf). One major mechanism of AMR in Gram-positive and mycobacterial pathogens affects three broad classes of front-line antibiotics — macrolides, lincosamides, and streptogramins (MLS) — and is conferred by a family of horizontally and vertically transmitted genes that encode the so-called erythromycin resistance methyltransferases (Erms) (2, 3, 4, 5, 6). Using SAM as a cofactor, Erms transfer a methyl group to adenosine at position 2058 (A2058) in the ∼3000 nucleotide-long 23S ribosomal RNA. This subtle posttranscriptional modification blocks the antibiotic-binding site in the ribosomal peptide exit tunnel (7). Horizontally transferred Erms are emerging in methicillin-resistant *Staphylococcus aureus* and vancomycin-resistant enterococci, for example, whereas endogenous, inducible Erms obviate the use of MLS antibiotics for *Mycobacterium tuberculosis* and limit their utility for *Mycobacterium abscessus* (5, 6, 8, 9, 10). Even at the level of pediatric acute otitis media caused by *S. aureus*, nearly 90% of clinical isolates in one study possessed Erms A, B, and C (11), which greatly limits available antibiotics in patients with β-lactam allergies.

Given the importance of Erms in MLS resistance, it is not surprising that there is significant genetic information about the diversity of types and distributions of Erms. The amino acid sequences and lengths can be broadly distinguished in mycobacteria and Gram-positive bacteria (12). Mycobacterial *erm* genes, such as *erm37* in *M. tuberculosis*, *erm38* in *Mycobacterium smegmatis*, and *erm41* in *M. abscessus*, as well as *ermE* of *Saccharopolyspora erythraea*, are genomically encoded, whereas the *erm* genes of Gram-positive bacteria, including *ermA* and *ermC* of methicillin-resistant *S. aureus* and *ermB* of vancomycin-resistant enterococci, are found on highly mobile plasmids involved in horizontal gene transfer. In terms of size, Erms A, B, and C share similar molecular weights (∼30 kDa), whereas Erm37 and Erm41 are smaller (∼20 kDa) because of the lack of a C-terminal domain (6, 10). These trends are broken by Erm38 and ErmE (∼45 kDa), which possess a longer C-terminal region that is predicted to be disordered (10, 13).

Despite the role of Erms in AMR, there is little structural information about the most clinically important Erms. Several Erm structures are available for Gram-positive bacteria, namely ErmAM (PDB access code: 1YUB) (14), ErmC (PDB access code: 1QAO) (15, 16), and ErmE (PDB access code: 6NVM) (13). However, there is no structural information for any mycobacterial Erm proteins, even though Erms confer intrinsic and inducible MLS resistance in mycobacteria (5), and Erm41 acquisition is limiting the efficacy of clarithromycin for *M. abscessus* infections (6, 17). Here, we determined the crystal structure of Erm38 of *M. smegmatis* and analyzed its biochemical and biophysical properties. Mutagenesis data and computational tools enabled us to build an atomic model for the Erm38-RNA complex, which will aid future drug discovery efforts.

## Results

### Overall structure of Erm38

Erm38 comprises 386 amino acids (NCBI WP_063844518; Uniport Q79N53), with analysis using the Database of Disordered Protein Predictions (18) predicting that the N-terminal 12 residues and C-terminal 124 residues are disordered regions. Expressing full-length Erm38 in *Escherichia coli* resulted in a heterogenous population of partially degraded proteins with molecular weights ranging from 30 to 45 kDa, in spite of length variations (Fig. S1). The computational prediction of a long, unstructured C terminus in Erm38 may account for this translational difficulty. We therefore expressed a truncated version of Erm38 spanning residues 13 to 261. This truncated Erm38, referred to as Erm38 throughout, has a molecular mass of 30 kDa and a size similar to most Erms from Gram-positive bacteria (Figs. S2–S4). The highly purified Erm38 formed square plate crystals (Fig. S5) that diffracted to a maximum resolution of 1.9 Å and tolerated up to 20% dimethylsulfoxide, which makes them suitable for ligand-soaking experiments. Hence, cocrystals with either SAM or the isosteric sinefungin inhibitor were obtained by soaking into native Erm38 crystals, and diffraction data to 2.25 Å resolution were collected as summarized in Table 1.

**Table 1:** Crystallographic data collection and refinement statistics

Parameter	Erm38 unliganded	Erm38 + SAM	Erm38 + SFG
Wavelength (Å)	0.98	0.98	0.98
Resolution^a^ range	21.54–1.9 (1.968–1.9)	19.94–2.25 (2.33–2.25)	19.91–2.25 (2.33–2.25)
Space group	P 4_2_ 2_1_ 2	P 4_2_ 2_1_ 2	P 4_2_ 2_1_ 2
Unit cell (Å, °)	77.663 77.663 101.116 90 90 90	78.02 78.02 101.54 90 90 90	78.018 78.018 101.536 90 90 90
Total reflections	657923 (66731)	400975 (34914)	400950 (34914)
Unique reflections	25025 (2449)	15428 (1483)	15427 (1483)
Multiplicity	26.3 (27.2)	26.0 (23.5)	26.0 (23.5)
Completeness (%)	99.88 (100.00)	99.64 (98.87)	99.64 (98.80)
Mean I/sigma(I)	30.08 (2.63)	34.02 (4.31)	34.01 (4.31)
Wilson B-factor (Å^2^)	37.56	40.72	42.93
R-merge^b^	0.06656 (1.321)	0.07489 (0.7235)	0.07489 (0.7235)
R-meas^c^	0.06792 (1.346)	0.07639 (0.7395)	0.07639 (0.7395)
R-pim^d^	0.01331 (0.2561)	0.01491 (0.1506)	0.01491 (0.1506)
CC1/2	1 (0.838)	1 (0.926)	1 (0.926)
CC	1 (0.955)	1 (0.981)	1 (0.981)
Reflections used in refinement	25022 (2449)	15422 (1483)	15423 (1482)
Reflections used for R-free	1279 (129)	758 (72)	758 (72)
R-work^e^	0.1921 (0.2501)	0.1882 (0.2390)	0.1942 (0.2398)
R-free^f^	0.2184 (0.2950)	0.2262 (0.3226)	0.2267 (0.3071)
CC(work)^g^	0.958 (0.842)	0.961 (0.874)	0.956 (0.829)
CC(free)	0.962 (0.723)	0.927 (0.764)	0.932 (0.850)
Number of nonhydrogen atoms	2078	2092	2047
Macromolecules	1906	1924	1885
Ligands		16	35
Solvent	172	152	127
Protein residues	245	246	244
RMS (bonds) (Å)	0.013	0.014	0.013
RMS (angles) (°)	1.55	1.75	1.63
Ramachandran favored (%)	99.16	98.35	98.32
Ramachandran allowed (%)	0.84	1.24	1.26
Ramachandran outliers (%)	0.00	0.41	0.42
Rotamer outliers (%)	3.12	4.21	4.76
Clashscore	0.52	2.05	1.81
Average B-factor (Å^2^)	43.44	45.91	47.23
Macromolecule	42.50	45.24	46.49
Ligand		63.04	65.08
Solvent	53.87	52.66	53.23

a The values presented in parentheses are for the highest resolution shell.

b $R_{merge}=\sum |I_{obs}-I_{avg}|/\sum I_{avg}$.

c $R_{meas}=\sum hkl\sqrt{\frac{n}{n-1}}\overset{n}{\underset{i=1}{\sum }}|I_{i}\left(hkl\right)-\bar{I}\left(hkl\right)|/\;\underset{hkl}{\sum }\overset{n}{\underset{i=1}{\sum }}I_{i}\left(hkl\right)$, where R_meas_ is the precision indicator of individual observation in unmerged data.

d $R_{pim}=\;\sum hkl\sqrt{\frac{1}{n}-1}\overset{n}{\underset{i=1}{\sum }}|I_{i}\left(hkl\right)-\bar{I}\left(hkl\right)|/\;\underset{hkl}{\sum }\overset{n}{\underset{i=1}{\sum }}I_{i}\left(hkl\right)$, where R_pim_ is the precision indicator of the merged data.

e $R_{work}=\sum |F_{obs}-F_{calc}|/\sum F_{obs}$.

f $R_{free}=\sum |F_{obs}-F_{calc}|/\sum I_{calc}\;$, where R_free_ was randomly sampled from 5% reflections.

g $Correlation\;Coefficient,\;CC=\;\sum \left(x-\langle x\rangle \right)\left(y-\langle y\rangle \right)/{\left[\sum {\left(x-\langle x\rangle \right)}^{2}\sum {\left(y-\langle y\rangle \right)}^{2}\right]}^{1/2}$, where CC_(1/2)_ is of two half datasets.

**Figure 1:**
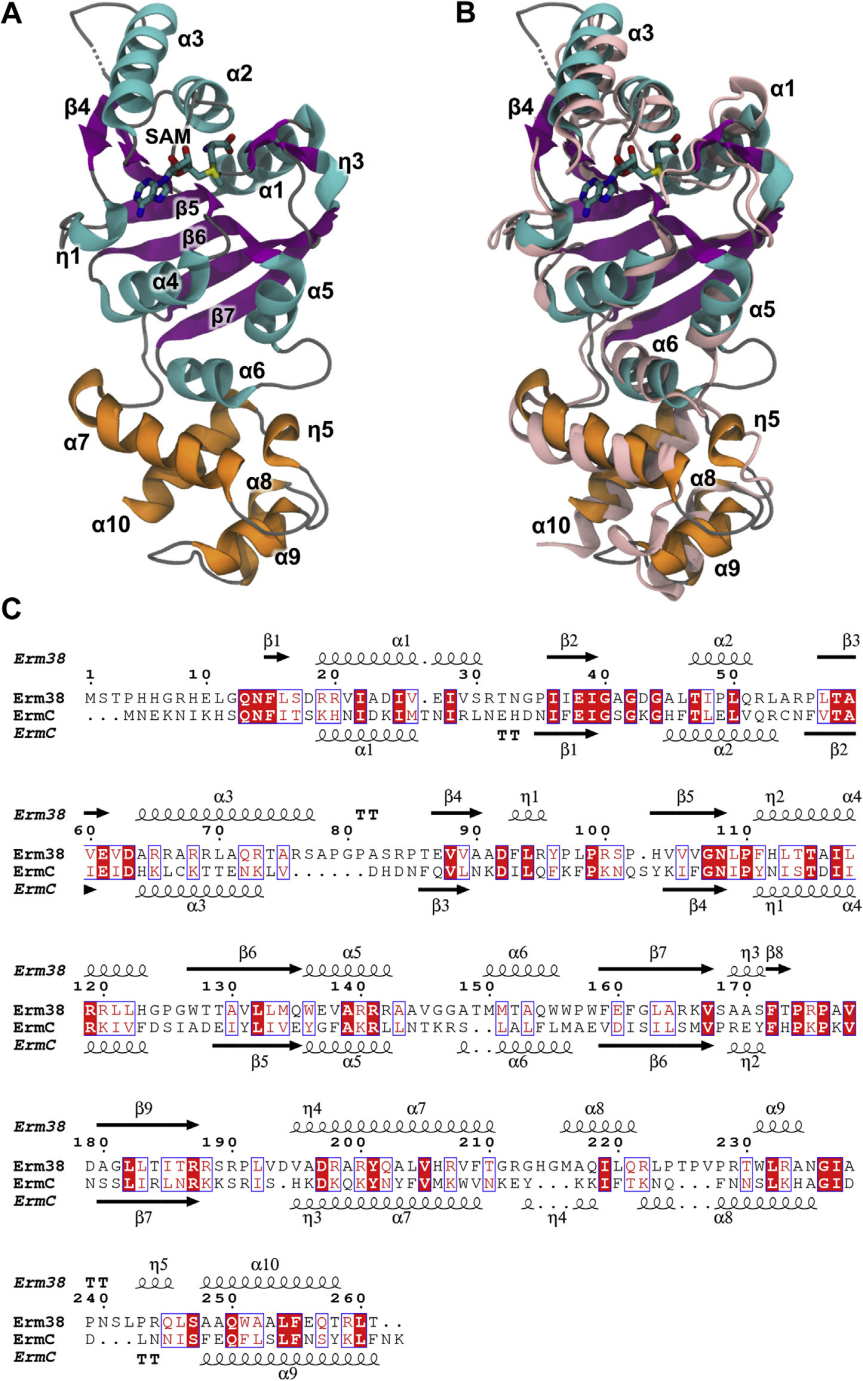
**Comparison between Erm38 and ErmC.***A*, crystal structure of Erm38 liganded with SAM. The β-sheets are colored in *purple*, helices at the N- and C-terminal are colored in *cyan* and *orange*, respectively. *B*, structural overlay of Erm38 and ErmC shown in *light pink ribbon*. *C*, sequence alignment of Erm38 and ErmC. Conserved residues are highlighted with *white text* on *red background*, and similar residues are shown in *red text*. The alignment was carried out with Clustal Omega (48) and visualized with ESPript 3.0 (49). Erm, Erythromycin resistance methyltransferase.

Erm38 features at its N-terminal catalytic domain the classic Rossmann fold, an α/β sandwich that contains the SAM cofactor-binding site and a helical C-terminal domain (Fig. 1*A*). Generally, Erm38 shares highly a similar structural fold with all published Erm structures and KsgA, a 16S rRNA methyltransferase (Fig. S6). A comparison between Erm38 and ErmC (Fig. 1*B*) reveals that both structures are highly similar with RMSD of 2.3 Å for 237 superimposed alpha carbon atoms, despite sharing only 22% sequence identity. Compared to ErmC, the N-terminal region of Erm38 has a longer α1 helix and longer loops connecting α3–β4 and α5–α6. In the C-terminal helical domain, Erm38 possesses a longer α8 helix but shorter α9 and α10 helices, along with an extra 3_10_ helix-labeled η5.

### Cofactor recognition at the SAM-binding pocket

**Figure 2:**
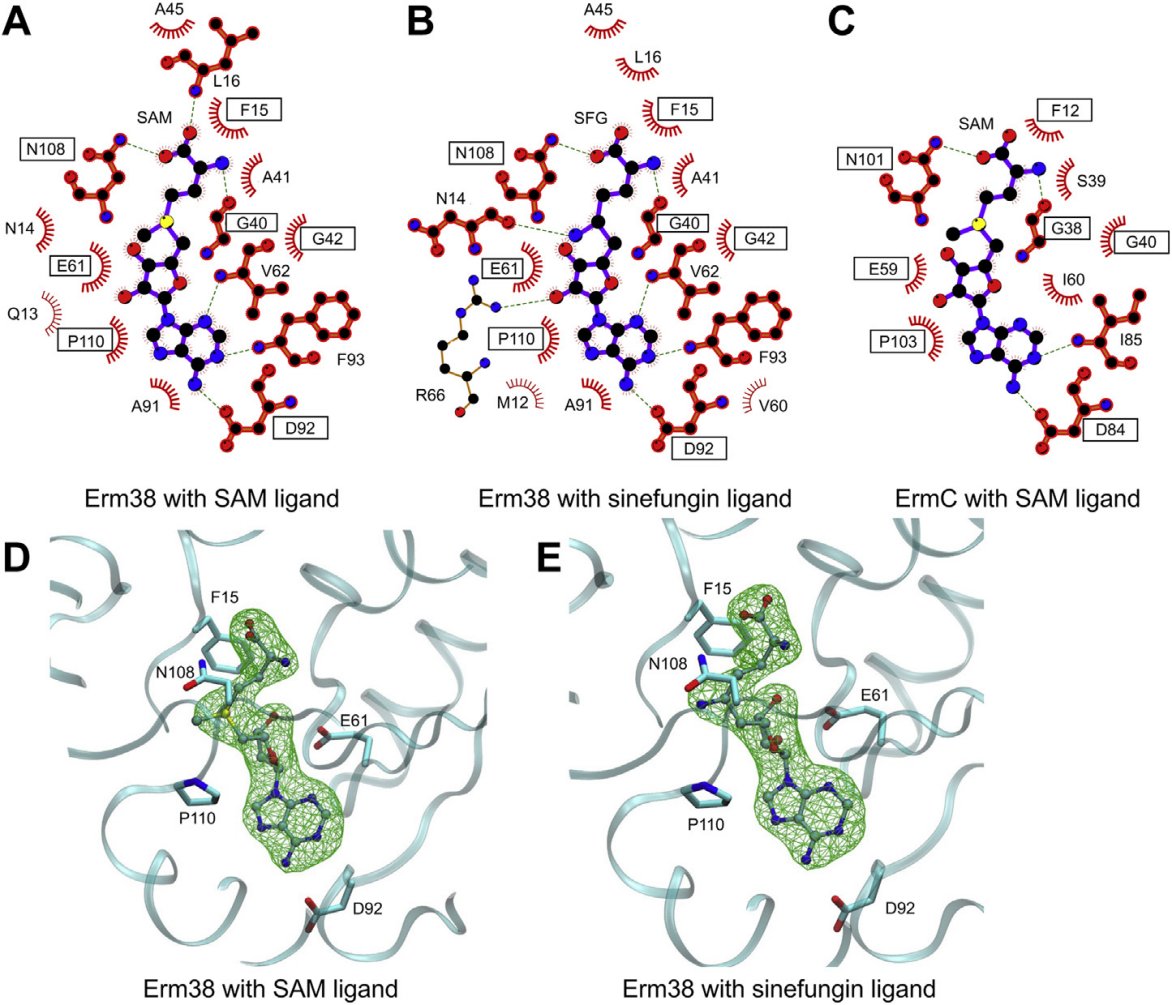
**2D and 3D representation of the protein-ligand interactions at the SAM-binding pocket.***A*–*C*, the 2D diagram was produced using Ligplot+ v2.2 (19). The *broken lines* indicate hydrogen bonds and salt-bridges. The residues involved in van der Waals interactions with the ligand are also shown. The *red crescents with the bristles* represent the hydrophobic interactions. Equivalent side chains are shown in *bold*, and strictly conserved residues are highlighted in *black boxes*. *D* and *E*, unbiased omit Fo-Fc electron density maps of the ligands contoured at 4 σ. Conserved residues F15, E61, D92, N108, and P110 were shown to facilitate cross-referencing with the 2D interaction diagram. Erm, Erythromycin resistance methyltransferase.

When analyzed using LigPlot (19), the binding of SAM and sinefungin inhibitor had almost identical interaction networks, with sinefungin forming an additional hydrogen bond with R66 (Fig. 2*B*). This was expected given the high chemical homology between SAM and sinefungin. The SAM-binding pocket of Erm38 was also compared with ErmC (Fig. 2, *A* and *C*). Despite sharing only 22% sequence identity, residues at the SAM-binding pocket are highly conserved between Erm38 and ErmC proteins with minor differences found at V62/I60, A41/S39, and F93/I85, whereas residues F15, G40, G42, E61, D92, N108, and P110 are identical for the two enzymes. Particularly, G40, G42, E61, D92, and P110 of Erm38 are strictly conserved among Erms A, B, C, E, 37, 38, and 41 (Fig. S7), suggesting an important functional role conserved during evolution.

### Erm38 methylation activity

Erm38 in *M. smegmatis* confers resistance to lincosamides and macrolides, but not streptogramin B antibiotics, which suggested that it was not a dimethyltransferase that produces double methylation at position N6 on A2058 (m66A) to cause full MLS (20). However, mass spectrometry-based methylation analysis revealed that Erm38 is indeed a dimethyltransferase, though with apparent low efficiency that results in mainly unmethylated and monomethylated sites (20). We thus compared Erm38 enzyme activity to Erms A, B, and C with the 32-mer RNA substrate and SAH production as the end point. As shown in Table 2 and Fig. S8, the Michaelis–Menten kinetic constants for Erm38 are very similar to those for the other Erms, which suggests that the “reluctance” observed by Madsen *et al.* involves factors other than the innate methylation activity, such as levels of enzyme induction following antibiotic exposure, availability of SAM, or accessibility of A2508 in the 23S rRNA.

**Table 2:** Michaelis–Menten kinetic parameters of various Erm enzymes

Enzymes	Km (μM)	V_max_ (nM/min)
ErmA	5.9 ± 0.8	84 ± 6
ErmB	5.5 ± 0.6	93 ± 4
ErmC	5.1 ± 1.0	90 ± 9
Erm38	6.8 ± 0.6	124 ± 6

### Putative RNA-binding site

**Figure 3:**
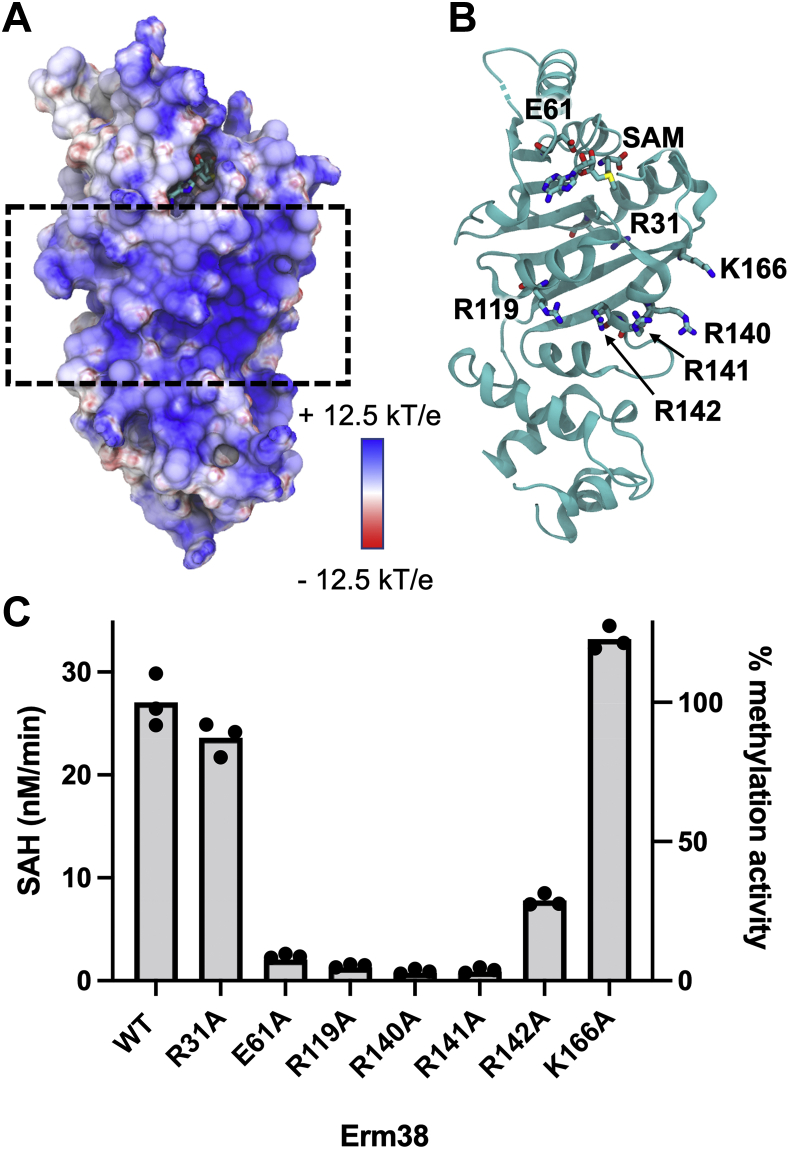
**Identification and validation of the residues that form the putative RNA-binding site.***A*, the putative RNA-binding site was identified by the highly positively charged region shown in the electrostatic potential of Erm38, which was calculated using APBS (50). *B*, positively charged residues in the putative RNA-binding site are identified and subjected to mutagenesis study. *C*, the methylation activity of Erm38 with various single mutations. The methylation assay was carried out in technical triplicates. Erm, Erythromycin resistance methyltransferase.

The electrostatic potential of Erm38 includes a highly positively charged region that comprises residues R119, R140, R141, R142, and K166 located at the protein surface (Fig. 3, *A* and *B*). As these solvent-exposed basic residues are likely to be involved in binding the RNA substrate, we produced an alanine mutant for each site and compared the enzymatic activities of the mutant and WT enzymes. R31A was selected as a negative control based on the fact that it is distant from the SAM- and RNA-binding sites, and we concluded that it does not participate in any intramolecular interaction needed to maintain the fold of the protein. E61K is known to reduce methylation activity by disrupting the SAM-binding site (21), so we selected this mutant as a control for loss of enzyme activity. As shown in Figure 3*C*, the E61K, R119A, R140A, and R141A mutations resulted in >90% reduction in methylation activity, whereas the R142A mutation resulted in 75% loss of enzymatic activity. Conversely, R31A and K166A did not affect the enzyme activity. Owing to their location at the surface of the protein and the absence of contact with SAM, the complete loss in activity observed upon loss of R119, R140, R141, and R142 is likely due to a disruption of electrostatic interactions with the RNA substrate.

**Figure 4:**
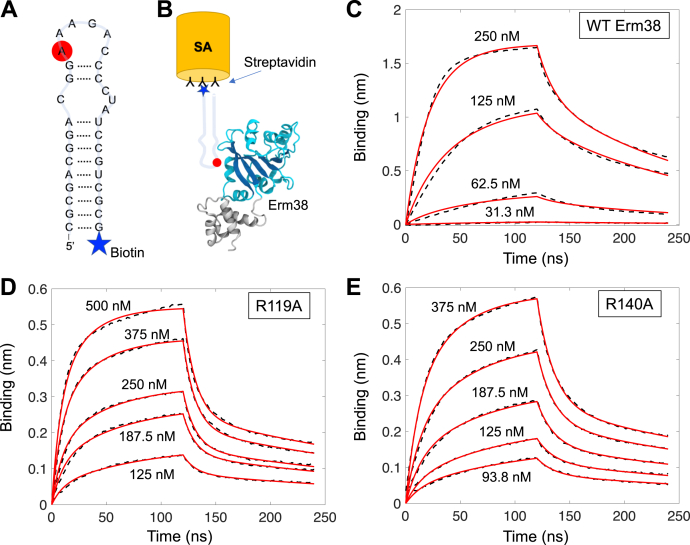
**Affinity binding curves of WT and single mutants of Erm38.** Using Biolayer Interferometry on a BioForte Octet Red96 system, association and dissociation rates were determined by immobilizing biotinylated-32mer RNA substrate (*A*) onto streptavidin biosensors (*B*), which will then be bound by the Erm38 proteins (*B*). The RNA-bound biosensors were incubated with specific concentrations of WT (*C*), R119A (*D*), and R130A (*E*) of Erm38 for 120 s to allow association. The sensors were then moved to protein-free solution and allowed to dissociate over 120 s. Curve fitting using a 2:1 interaction model allows for the affinity constants to be measured. Erm, Erythromycin resistance methyltransferase.

We next sought to quantify the effect of the mutations on the interaction between Erm38 and its RNA substrate, using biolayer interferometry to measure the kinetics of protein binding to a surface-bound biotinylated 32-mer RNA substrate (Fig. 4*A*). Surprisingly, the RNA-binding profiles for WT and mutants were similar, with an interaction model was best represented by 2:1 heterogeneous binding and two dissociation constants (K_D_) (Table 3). The micromolar K_D2_ values likely represent a small amount of nonspecific binding, whereas the higher affinity K_D1_ of around 50 nM represents the binding affinity of Erm38 for its RNA substrate.

**Table 3:** Octet kinetic fit values for the WT and mutants of Erm38 using 2:1 heterogenous ligand fitting model

Enzymes	K_d_1 (nM)	K_on_1 (1/M∗s)	K_dis_1 (1/s)	K_d_2 (nM)	K_on_2 (1/M∗s)	K_dis_2 (1/s)
Erm38 WT	45.0 ± 1.5	1.26 ± 0.03 × 10^5^	5.68 ± 0.13 × 10^−3^	3742 ± 687	1.76 ± 1.73 × 10^4^	6.59 ± 0.32 × 10^−2^
Erm38 R119A	50.3 ± 1.3	6.46 ± 0.09 × 10^4^	3.25 ± 0.07 × 10^−3^	1258 ± 17	7.14 ± 0.30 × 10^4^	8.98 ± 0.10 × 10^−2^
Erm38 R140A	58.8 ± 2.2	5.33 ± 0.11 × 10^4^	3.14 ± 0.10 × 10^−3^	1241 ± 23	5.27 ± 0.31 × 10^4^	6.54 ± 0.10 × 10^−2^

### Structural model for the Erm38–RNA complex

Guided by the mutagenesis data and the knowledge that Erm38 binds strongly to the 32-mer RNA substrate, we created an atomic model of the Erm38–RNA complex. We first performed molecular docking of the RNA substrate on Erm38. Of 100 docked structures, one was most consistent with the electrostatic potential analysis and mutagenesis data, with adenine at position 13 (equivalent to A2058 in *E. coli* 23S rRNA) lying in the vicinity of F111 of Erm38, an aromatic residue known to interact with the adenine to facilitate methylation (21).

**Figure 5:**
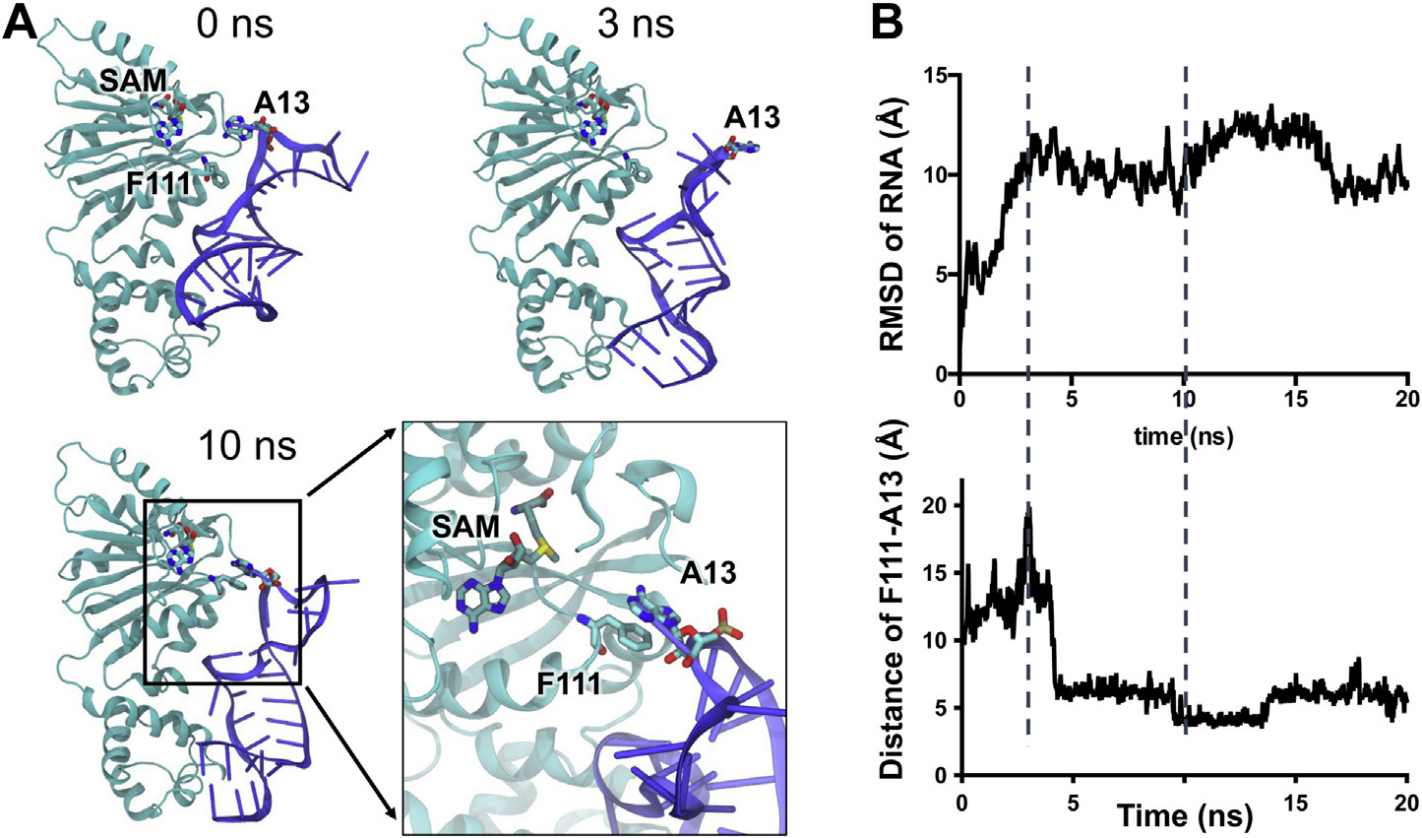
**Molecular dynamics simulation of the Erm38+RNA complex.***A*, the protein–RNA interaction was refined where the RNA substrate undergoes a conformational change to bind to the C-terminal of Erm38, and a π-π stacking interaction is formed between F111 of Erm38 and A13 of RNA. Note that A13 is equivalent to A2058 in *E. coli* 23S ribosomal RNA numbering. *B*, trajectories of the RMSD values of the RNA substrate and the distance between F111 and A13 over the 20 ns of simulation. Erm, Erythromycin resistance methyltransferase.

The docked structure was then subjected to molecular dynamics (MD) simulation to further refine the binding interface. Figure 5 shows a significant rearrangement of the RNA substrate in the first 10 ns of the simulation. At 3 ns, the stem loop of the RNA substrate relaxes and extends to form tighter interactions with the C-terminal domain of Erm38, whereas the A13 methylation site extends away from the catalytic site. From 5 ns onwards, the A13 reinserts into the catalytic pocket by the formation of a stable π-π stacking interaction with the phenyl ring of F111. After the initial refinement of the binding interface, the RMSD value of the RNA substrate remains at ∼10 Å, indicating that the system has reached an equilibrium.

**Figure 6:**
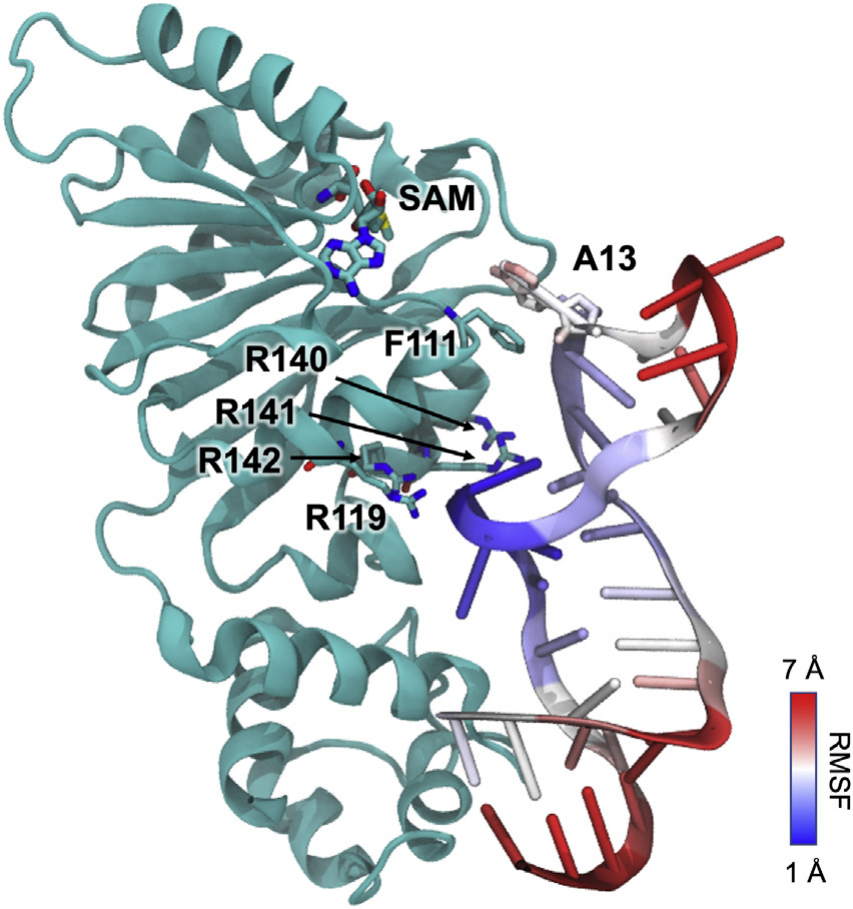
**The four arginine residues bind stably to the RNA substrate.** The degree of fluctuation of the RNA substrate was measured in the course of simulations by root-mean-square fluctuation (RMSF). The residues in *red* map to regions with relatively large fluctuation (∼7 Å), whereas those colored *blue* map to regions with small fluctuation (∼1 Å). The *dark blue region* at the middle segment of the RNA substrate highlights the strong RNA binding of the R119, R140, R141, and E142 of Erm38. Erm, Erythromycin resistance methyltransferase.

By analyzing the root-mean-square fluctuation of the RNA substrate (Fig. 6), we found that the portion of the RNA substrate that interacts with the patch of positively charged residues is most stable, validating the importance of these surface-exposed residues for RNA binding. In contrast, the A13-containing stem loop and the substrate termini remain relatively flexible. From the pool of 100 docked structures, we also simulated another docked Erm38-RNA model that is closest to the relative orientation of protein-RNA observed in KsgA-RNA model (PDB accession number 3FTF). The Erm38–RNA complex is stable in the 10-ns simulation, whereas this alternate model lacks the π-π stacking interaction and it does not interact with R140 of Erm38, which was shown to be important according to our mutagenesis data (Fig. S9). Therefore, the structural model of Erm38 complexed with the RNA substrate shown in Figures 5 and 6 provides the most accurate view of how Erm binds to its RNA substrate.

## Discussion

**Figure 7:**
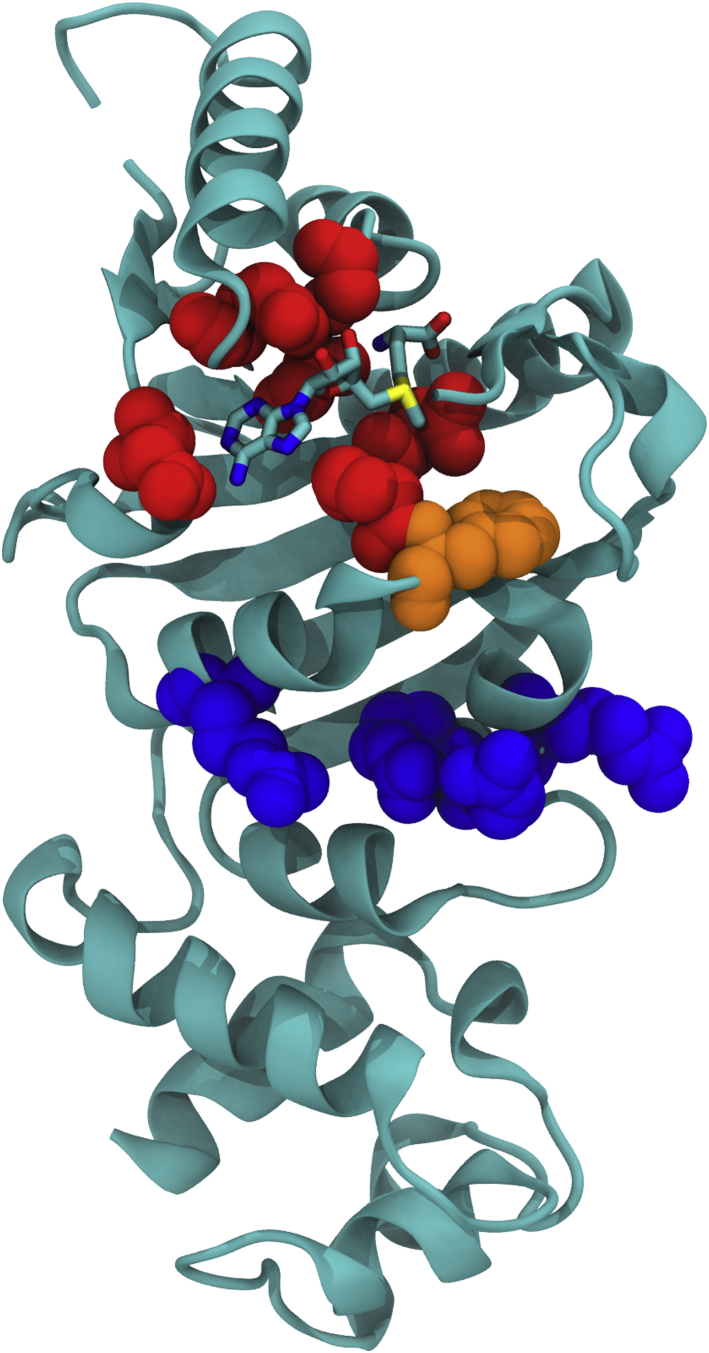
**Erm38 structure depicting the locations of key spatially conserved amino acids at the SAM-binding site (*red*), catalytic site (*orange*), and RNA-binding site (*blue*).**Table 4 shows the proximity of these conserved amino acids in Erms AM, C, and E. Erm, Erythromycin resistance methyltransferase.

Despite sharing low amino-acid sequence identity with the other Erms whose structures were reported previously (13, 14, 15, 16), Erm38 shares high structural similarity. This conclusion is validated by DALI distance-matrix structural homology searches (22) against Erm38, which return high Z value scores of 28 for ErmE, 24 for ErmC, and 16 for ErmAM. The particularly high score with ErmE is not surprising because ErmE and Erm38 are both from actinomycetes and both have long unstructured C-termini. Considering that the N-terminal region of Erm38 shares 30% sequence identity and over 40% sequence similarity with Erm37 in *M. tuberculosis* and Erm41 in *M. abscessus* (Fig. S10), two high-value therapeutic targets, the structure of Erm38 from *M. smegmatis* reported here provides a critical foundation on which to build accurate structural models for the other mycobacterial Erms for drug design and discovery. One striking feature of all Erms is the high degree of spatial conservation of specific amino acids in the SAM-binding, RNA-binding, and catalytic domains as detailed in Table 4 and depicted structurally in Figure 7.

**Table 4:** Conserved spatial locations of conserved amino acids in Erms

Location in Erm	Erm38		ErmAM			ErmC			ErmE		
	AA	#	AA	#	Δ, Å^a^	AA	#	Δ, Å	AA	#	Δ, Å
SAM binding	GLY	40	GLY	37	3.9	GLY	38	0.8	GLY	69	1.5
	GLY	42	GLY	39	2.5	GLY	40	1.2	GLY	71	2.1
	GLU	61	GLU	58	1.3	GLU	59	1.6	GLU	90	1.1
	ASP	92	ASP	83	3.2	ASP	84	1.7	ASP	115	0.5
	ASN	108	ASN	100	2.9	ASN	101	0.9	ALA	131	0.7
	PRO	110	PRO	102	1.4	PRO	103	1.5	PRO	133	1.0
Catalytic	PHE	111	TYR	103	1.6	TYR	104	1.8	TYR	134	1.2
RNA binding	ARG	119	LYS	111	3.0	ARG	112	2.2	ASP	142	1.4
	ARG	140	LYS	132	3.5	LYS	133	1.3	ARG	163	0.4
	ARG	141	ARG	133	3.6	ARG	134	0.9	LYS	164	0.4
	ARG	142	THR	134	2.5	LEU	135	1.1	ARG	165	0.8

a Δ, Å: C_α_-C_α_ distance in Angstroms.

As our mutagenesis experiments demonstrated, the amino acids involved in target recognition by Erm38 remain elusive. Although several positively-charged sites proved to be essential for methylation activity, mutation of these sites individually did not cause significant changes in binding affinity for the RNA substrate. Such a disconnect between enzyme activity and RNA-binding affinity was observed in ErmE (23) and is analogous to the lack of correlation between binding affinity and specificity for aptamers (24). Maravic *et al.* (25) also described an *E. coli* strain carrying ErmC gene with a R134A mutation (equivalent to R141A of Erm38) that resulted in complete loss of erythromycin resistance, whereas mutations R112A and K133A in ErmC (equivalent to R119A and R140A of Erm38) reduced erythromycin resistance. These three amino acid positions are highly conserved across all seven Erms compared in this study (Fig. S7 and Table 4). Taken together, these observations suggest a model in which a redundancy of patches of basic residues confer high-affinity binding, whereas other yet-to-be-defined protein-RNA contacts, such as stacking between F111 and A2058, confer the target recognition specificity for A2058.

One reason for the paucity of information about Erm-RNA structures is the adventitious precipitation of the Erm–RNA complex when the 32-mer RNA substrate is mixed with the apoprotein. Such precipitation was also observed when using full length ErmB and ErmC (data not shown). This rules out X-ray crystallography, cryo-EM, and solution-state NMR techniques for structure determination. Solid-state NMR offers a solution to analyze the precipitate, whereas the 40 kDa size of the Erm38–RNA complex poses a problem for NMR resolution.

Here, we took the alternative approach of integrating biochemical data, unbiased molecular docking, and MD simulations to provide a plausible atomic model of the Erm38–RNA complex (Figs. 6 and 7). The resulting model suggests that, after RNA binding to the patch of positive-charged residues (Fig. 7), A13 of the 32-mer RNA undergoes π-π stacking with F111, an amino acid known to be essential for Erm38 catalytic activity (21). This π-π interaction that was organically formed in the simulation appears to be a key event that facilitates the methylation reaction. The Erm38-RNA model proposed here not only substantiates the important role of the positively charged residues at the N-terminal domain of Erm38 in RNA binding, but it also shows a binding contribution for the C-terminal region of Erm38. Specifically, highly conserved residues R207, R212, and R222 were found to form transient hydrogen bonds with the RNA substrate. That the C-terminal region of Erm plays a role in RNA binding and determines its methylation specificity is supported by the observations of Madsen *et al.* (10) with Erm37 of *M. tuberculosis*, which lacks a C-terminal domain and promiscuously methylates neighboring adenosines in the ribosomal RNA. The RNA-binding interactions of the conserved C-terminal residues observed in the simulation support the critical role of the C-terminal domain in determining the methylation activity of Erm proteins.

In conclusion, the crystal structure of Erm38 of *M. smegmatis* and the atomic model of Erm38 in complex with a 32-mer RNA substrate presented here can be used for structure-based drug design to target the putative RNA-binding site. Nonconventional design approaches such as creating compounds that mimic the footprint of the RNA substrate can now be tested. Furthermore, the high tolerance for dimethylsulfoxide of the Erm38 crystals obtained in this work enables hit identification and hit-to-lead optimization in following drug screening campaigns.

## Experimental procedures

### Expression and purification of Erm38 and its mutants

The *erm38* gene was synthesized and cloned into pNIC28-Bsa4 vector by Bio Basic Inc. Nucleotide sequences were codon-optimized to improve the efficiency of soluble expression in *E. coli*. The plasmid-containing truncated Erm38 (residues 13–261) was produced by the NTU Protein Production Platform (Singapore). The truncated Erm38 is referred to throughout as Erm38. Site-directed mutagenesis was performed on Erm38 to introduce the single mutations R31A, R119A, R140A, R141A, R142A, and K166A, using primers listed in Table S1. The mutation sites were confirmed by Sanger sequencing. After transformation into *E. coli* BL21(DE3)-T1R Rosetta strain, Erm38 proteins were expressed by growing in LB broth containing 34 μg/ml chloramphenicol and 50 μg/ml kanamycin at 37 °C, with expression induced with 0.2 mM IPTG for 18 h at 16 °C. Cell pellets were lysed by sonication in 50 mM Na Hepes pH7.5, 500 mM NaCl, 10% glycerol, 0.5 mM DTT, and 10 mM Imidazole. After removal of cell debris by centrifugation at 16,000*g*, the supernatant was sterile filtered and subjected to a series of purification steps. For WT Erm38, high-purity protein was critical for crystallization studies, which necessitated a three-step purification involving immobilized metal affinity chromatography (IMAC), cation-exchange chromatography, and size-exclusion chromatography (see Supplemental Methods). For the Erm38 mutants, a single-step IMAC purification was performed to obtain catalytically active proteins. The IMAC eluates were subjected to buffer exchange by diafiltration to reduce the imidazole concentration to <10 mM. All purified proteins were concentrated to >5 mg/ml and stored at −80 °C in 20 mM Hepes pH 7.5, 300 mM NaCl, 10% glycerol, and 2 mM DTT.

### Crystallization of Erm38

The purified WT Erm38 at 15 mg/ml was subjected to initial crystallization screening. The screen was set up using the sitting drop vapor diffusion method with Morpheus and JCSG-plus crystallization kits (Molecular Dimensions) on Intelli-plates 96-3 (Art Robbins Instruments) (26, 27, 28). Using a Mosquito HTS (TTP Labtech), crystallization trials were set with protein-to-reservoir volume ratios of 2:1, 1:1, and 1:2. Plates were sealed and kept at 20 °C in FORMULATRIX for monitoring the crystallization process. Small square crystals grew in 0.8 M sodium succinate pH 7.0. Upon further optimization, large square plate crystals grew in 1.0 M succinic acid and 5% glycerol within 5 days. To obtain the structure of Erm38 with SAM and sinefungin ligands, a few well-formed crystals were selected and soaked for 24 h with 2 mM SAM or 2 mM sinefungin. Before X-ray diffraction data collection, the crystals were protected from freezing damage using the corresponding crystallization buffer supplemented with 20% (v/v) glycerol before being flash-frozen in liquid nitrogen.

X-ray diffraction data were collected at 0.98 Å wavelength at the beamline PROXIMA 2A at the synchrotron SOLEIL (29, 30). The datasets were processed with the program XDS (31) and the CCP4 program (32). The structure was solved by molecular replacement with BALBES (33) using PDB ID 3FTF and 3FUV as search models. Several rounds of manual model building were then performed using program COOT (34), interspersed with structure refinement with programs REFMAC (35) and BUSTER (36). Some extra peaks in the Fo-Fc residual map at the SAM-binding site were not accounted for and left unmodeled in the liganded Erm38 models, as we only modeled the most occupied conformations of the SAM and sinefungin ligands. These unmodeled electron density suggested the possibility of an alternate conformation of the carboxylic tail. All figures representing structures were made using visual molecular dynamics (VMD) (37). Data collection and refinement statistics can be found in Table 1.

### Erm enzyme assay

All assays were performed in 10 μl total volume in a 384-well plate (Grenier, Item No. 784904; white color). Erm reaction kinetics were quantified using the MTase-Glo methyltransferase assay kit (Promega), which detects the SAH product of SAM demethylation in a coupled luminescence reaction (38). The RNA substrate for the reaction was a 32-mer oligoribonucleotide, CGCGACGGACGGAAAGACCCC UAUCCGUCGCG, which has been shown to be a universal Erm substrate (39, 40) that was designed to mimic the adenine loop in domain V of 23S rRNA with its methylation site confirmed by mass spectrometry (41). The RNA substrate was prepared by denaturing at 90 °C for 1 min and reannealing by cooling slowly to ambient temperature. Assays for evaluating the activity of various Erm38 mutants contained 2 μM Erm protein, 2 μM RNA substrate, and 20 μM SAM in a buffer comprised of 50 mM Na Hepes pH 7.5, 40 mM KCl, 1 mM MgCl_2_, and 1 mM DTT (25, 38, 39, 42). To determine the Michaelis–Menten kinetic constants, the RNA substrate was varied from 0.25 μM to 8 μM. The reactions were carried out by mixing RNA and SAM and adding Erm38 and MTase-Glo reagent. After a 30 min incubation at 37 °C, 5 μl of MTase-Glo detection solution was added to initiate the luminescence reaction. The luminescent signal was measured for 30 min using a microplate reader (BioTek Synergy 4 Plate Reader), with the resulting luminescent signal converted to SAH concentration using a SAH standard curve. A reaction without Erm protein was included as a control. The Michaelis–Menten kinetic parameters (maximum rate, V_max_; Michaelis constant, K_m_) were determined by plotting the SAH concentration as a function of RNA concentration tested and the data were fitted by nonlinear regression with GraphPad Prism version 9 (GraphPad Software, Inc).

### Biolayer interferometry to quantify protein–RNA interaction

The binding affinities of WT, R119A, and R130A Erm38 proteins with the 32-mer RNA substrate were measured by biolayer interferometry using an Octet RED96e (ForteBio), with binding data acquired (kinetics mode) and analyzed using built in software. Biosensors were hydrated in phosphate-buffered saline with Tween (PBST) buffer (137 mM sodium chloride, 2.7 mM potassium chloride, 12 mM phosphate, pH 7.4, 0.005% Tween 20, and 0.5 mg/ml bovine serum albumin) for 20 min at ambient temperature preceding data acquisition, and experiments were performed at 25 °C. Erm38 proteins were diluted in PBST buffer. A biotinylated 32-mer RNA substrate was synthesized (Sigma-Aldrich) with biotin appended at the 3′-end of the oligo. The biotinylated RNA was diluted to 100 nM and immobilized on a streptavidin-coated biosensor with a signal threshold of 0.2 nm. A baseline level was established for 60 s before Erm38 proteins were exposed to RNA-loaded biosensors for 120 s and dissociated in PBST buffer for 120 s. To determine K_d_ values for each Erm38 protein, a reference sensor with loaded RNA but no protein was subtracted from the data before fitting. Curve fitting using a 2:1 interaction model was used to measure the dissociation constant (K_D_) for WT and mutants of Erm38.

### Molecular docking and MD simulations

Secondary structure of the 32mer RNA substrate was predicted using RNAfold (43), and the 3D model of the 32-mer was constructed computationally using 3dRNA (44). Of the five models predicted by 3dRNA, the 3D model selected for docking has its A13 (equivalent to A2058 in *E. coli* numbering) pointing outwards, which makes this RNA model primed to interact with the methylation site of Erm. Docking of Erm38 and 32-mer RNA was performed using HDOCK (45) without any prior knowledge of the putative-binding site. Of 100 top docked structures, the Erm38 + 32-mer RNA model that best fits the mutagenesis data was selected.

The selected Erm38 + 32mer RNA complex was subjected to all-atom, explicit solvent MD simulation using NAMD (46). The complex was simulated in a water box, where the minimal distance between the solute and the box boundary was 15 Å along all three axes. The charges of the solvated system were neutralized with counter-ions, and the ionic strength of the solvent was set to 150 mM NaCl using VMD (37). The fully solvated system was subjected to conjugate-gradient minimization for 10,000 steps, subsequently heated to 310 K, and a 5 ns equilibration with protein and RNA backbone atoms constrained using a harmonic potential of the form U(x) = k (x − x_ref_)^2^, where k is 1 kcal mol^−1^ Å^−2^ and x_ref_ is the initial atom coordinates. Finally, 20 ns production simulations were performed without constraints. The simulation was performed under the NPT ensemble assuming the CHARMM36 force field (47) for the protein and RNA molecules and the TIP3P model for the water molecules. All simulation trajectory analysis including RMSD and root mean square fluctuation were performed using VMD.

## Data availability

Atomic coordinates and structure factors for the reported crystal structures have been deposited with the Protein Data bank under accession numbers 7F8A (unliganded Erm38), 7F8B (Erm38 complexed with SAM), and 7F8C (Erm38 complexed with SFG).

## Supporting information

This article contains supporting information.

## Conflict of interest

The authors declare that they have no conflicts of interest with the contents of this article.
