# Supplementary material for: Crystal structure and functional analysis of mycobacterial erythromycin resistance methyltransferase Erm38 reveals its RNA-binding site
Source: J Biol Chem. 2022 Jan 8;298(2):101571. doi: 10.1016/j.jbc.2022.101571 (PMC8844858; doi:10.1016/j.jbc.2022.101571)
Supplement: Supplemental Methods, Figures S1–S10 and Table S1 [file mmc1.pdf]

# Supporting Information for

# Crystal structure and functional analysis of mycobacterial Erm38 reveals its RNA binding site

Boon Chong Goh, Xinyu Xiang, Julien Lescar, and Peter C. Dedon

Contents:

• Supplemental Methods
• Figures S1-S10
• Table S1

## Supplementary methods

### Purification and analysis of Erm38

The following Erm38 construct was cloned in pNIC28-Bsa4 and expressed in *E. coli*. The sequence corresponds to residues Q13 to T261 in the full length Erm38.

```
MQNFLSDRRVIADIVEIVSRTNGPIIEIGAGDGALTIPLQRLARPLTAVEVDARRARRLAQRTA
RSAPGPASRPTEVVAADFLRYPLPRSPHVVVGNLPFHLTTAILRRLLHGPGWTTAVLLMQWEVA
RRRAAVGGATMMTAQWWPWFEFGLARKVSAASFTPRPAVDAGLLTITRRSRPLVDVADRARYQA
LVHRVFTGRGHGMAQILQRLPTPVPRTWLRANGIAPNSLPRQLSAAQWAALFEQTRLT
```

The crude cell content showed overexpressed protein in SDS-PAGE (**Figure S2a**). To purify the protein, a three-step purification was performed. The first purification employs immobilized metal affinity chromatography (IMAC) using Ni-NTA beads. The IMAC base buffer is 50 mM HEPES pH7.5, 500 mM NaCl, 10% glycerol. After IMAC purification, multiple bands were still present on SDS-PAGE (**Figure S2b**).

Ion exchange chromatography (IEX) was performed as the second purification step. The eluate was loaded onto HiTrap SP HP, a cation-exchange column and one single peak was observed around fractions 30-36, which is equivalent to 500mM-865mM of NaCl concentration (**Figure S3a**). SDS-PAGE analysis showed one major single band around 30kDa, suggesting relatively pure composition of the eluate (**Figure S3b**).

To remove the remaining trace of contaminants, size exclusion chromatography (SEC) was performed with eluate (fractions 30-36) from IEX, during which one single peak was observed at 96ml elution volume (**Figure S4a**). SDS-PAGE analysis showed one single band around 30kDa, consistent with that obtained from IEX purification (**Figure S4b**). The fractions containing the target protein Erm38 from the main peak (fractions 69-75) were pooled and concentrated to 15mg/ml for crystallization.

## Supplementary Figures and Tables

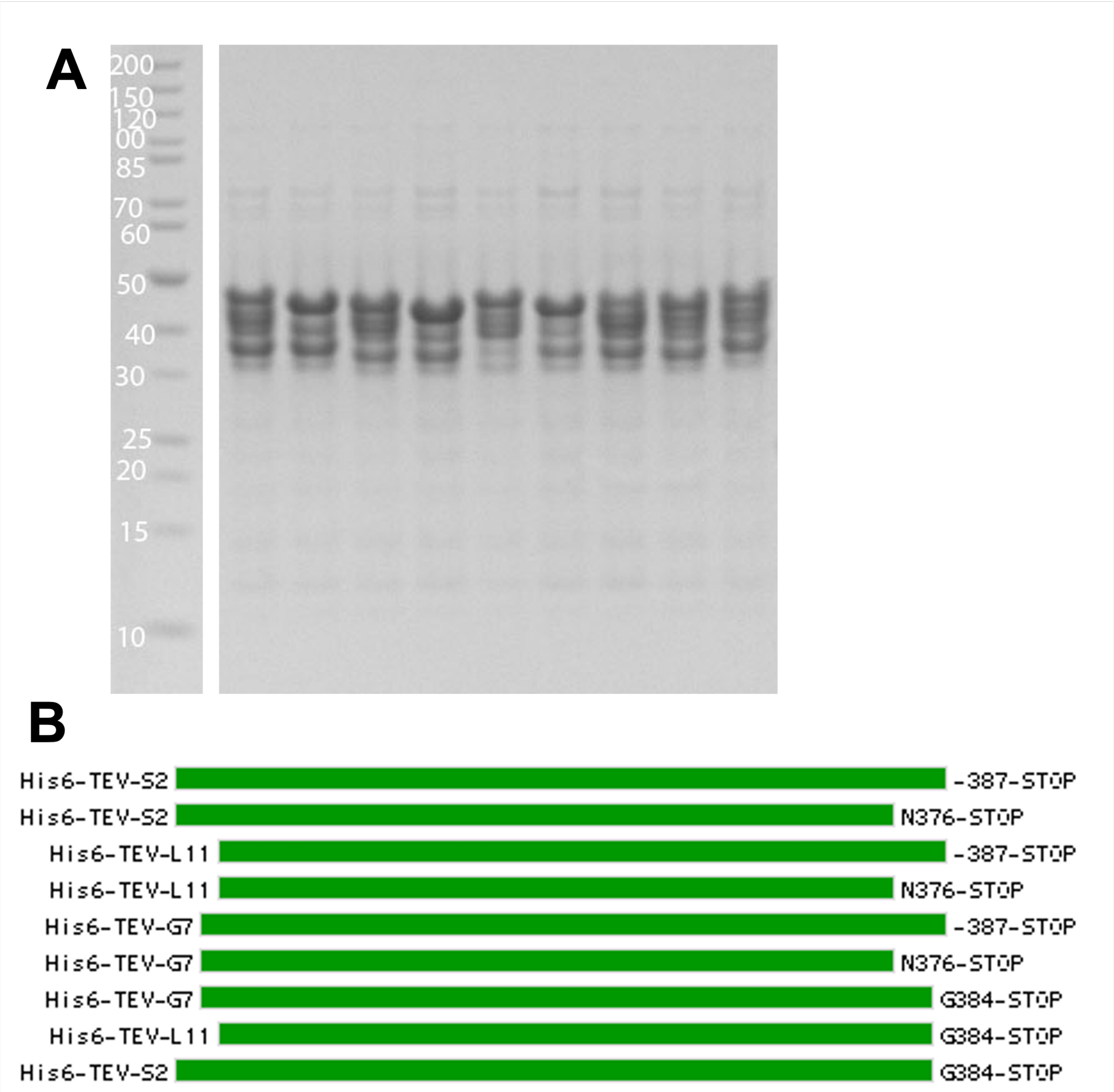


**Figure S1**. Heterogenous soluble expression for full length Erm38 with 386 amino acid residues. Several minor length variations were done to improve the expression but to no avail. Note that the SDS-PAGE image shown in A was obtained from the same gel with 3 irrelevant lanes between the ladder and lanes of interest removed.

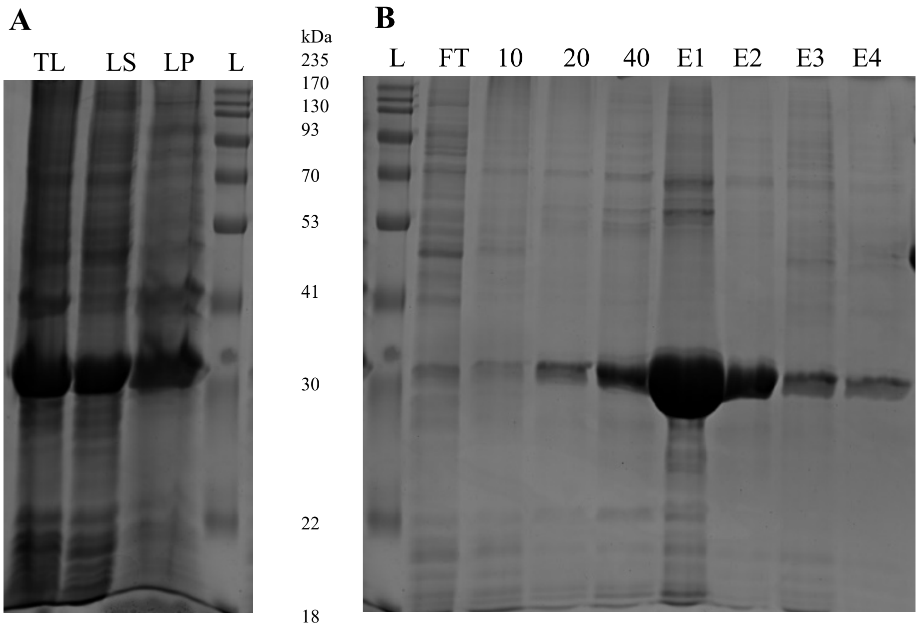


**Figure S2**. Coomassie-stained SDS-PAGE gel. (**A**) crude cell content after lysis. TL is total lysate; LS is lysate supernatant; LP is precipitate in lysate. L: Protein ladder. (**B**) Erm38 after IMAC purification. L: Protein ladder, FT is flow through, 10, 20 and 40 are wash samples with IMAC buffers with 10, 20, and 40 mM imidazole; E1-4 are eluates at 250 mM imidazole. The two protein ladders shown in (**A**) and (**B**) are identical.

A

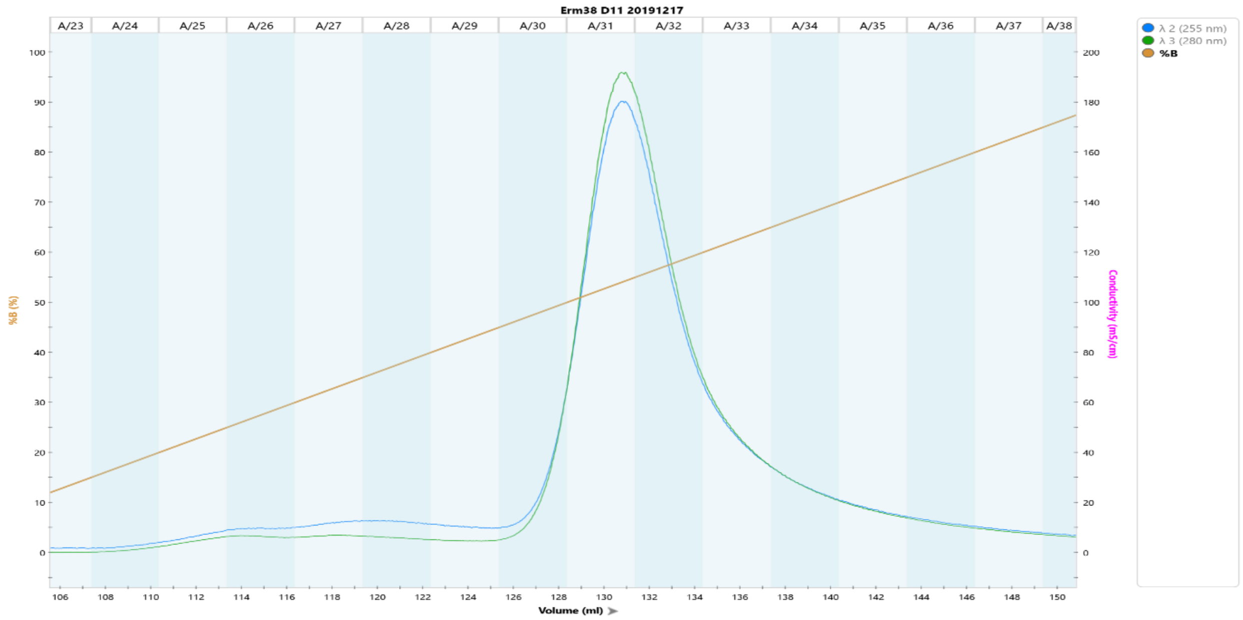


B

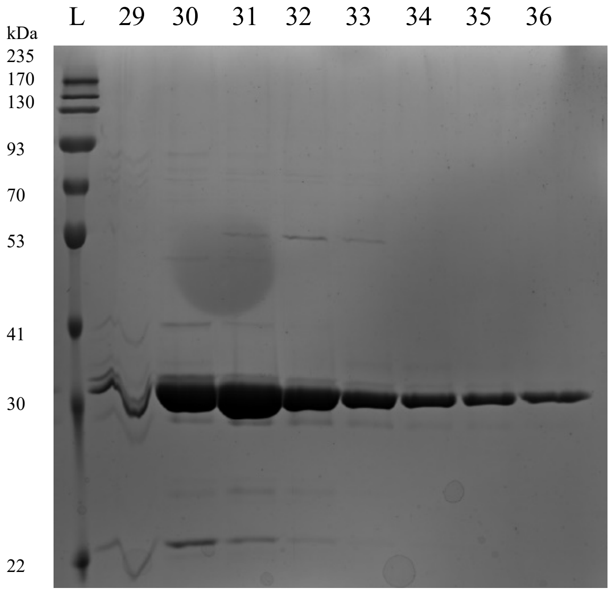


**Figure S3**. IEX purification of Erm38. (**A**) chromatogram of Erm38 elution under an increasing salt concentration gradient. (**B**) Coomassie-stained SDS-PAGE gel. L: Protein ladder. 29-36 are fractions in correspondence to the peak in chromatogram.

**A**

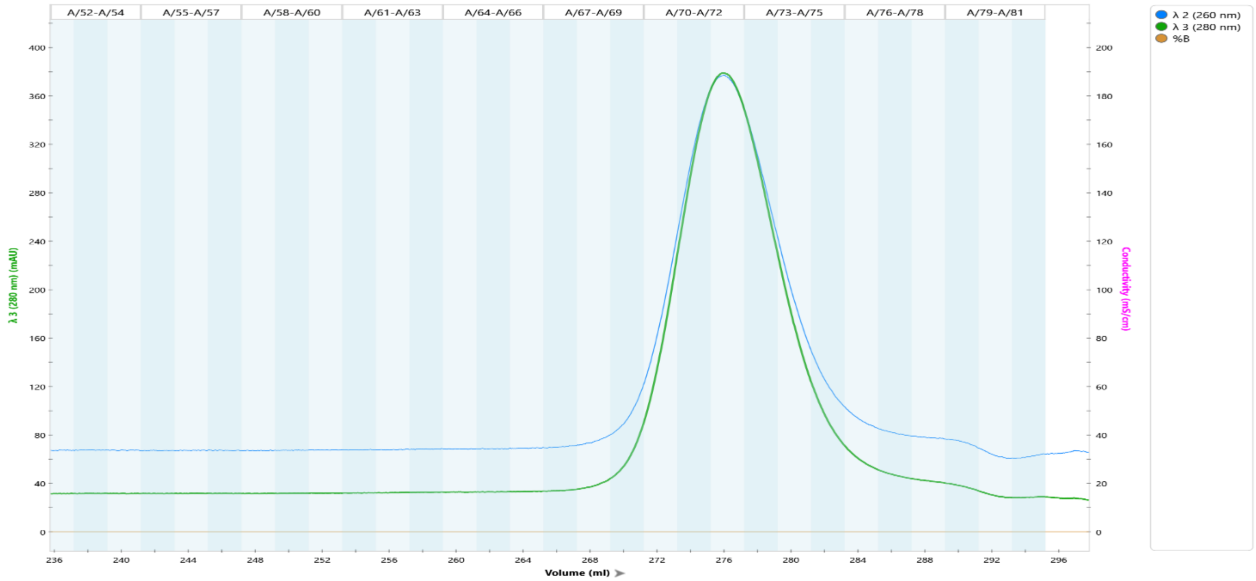


**B**

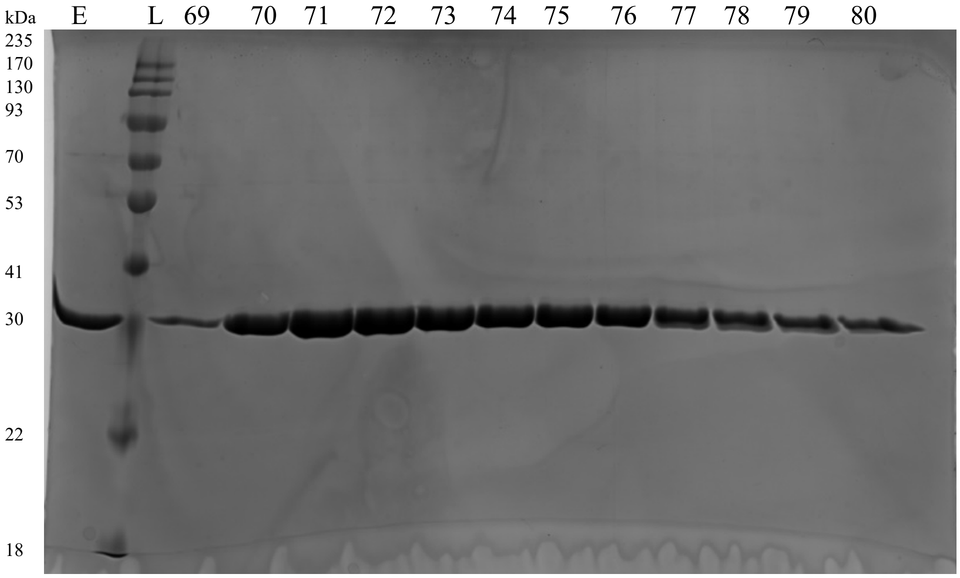


**Figure S4**. Size-exclusion chromatography (SEC) purification of Erm38. (**A**) Chromatogram of Erm38. The correct elution volume of the peak is 96ml. The volume axis showed in the figure wasn't reset before the start of SEC. (**B**) Coomassie-stained SDS-PAGE gel. E is eluate from IEX, before SEC; L is ladder; 69-80 are fractions in correspondence to the peak in chromatogram.

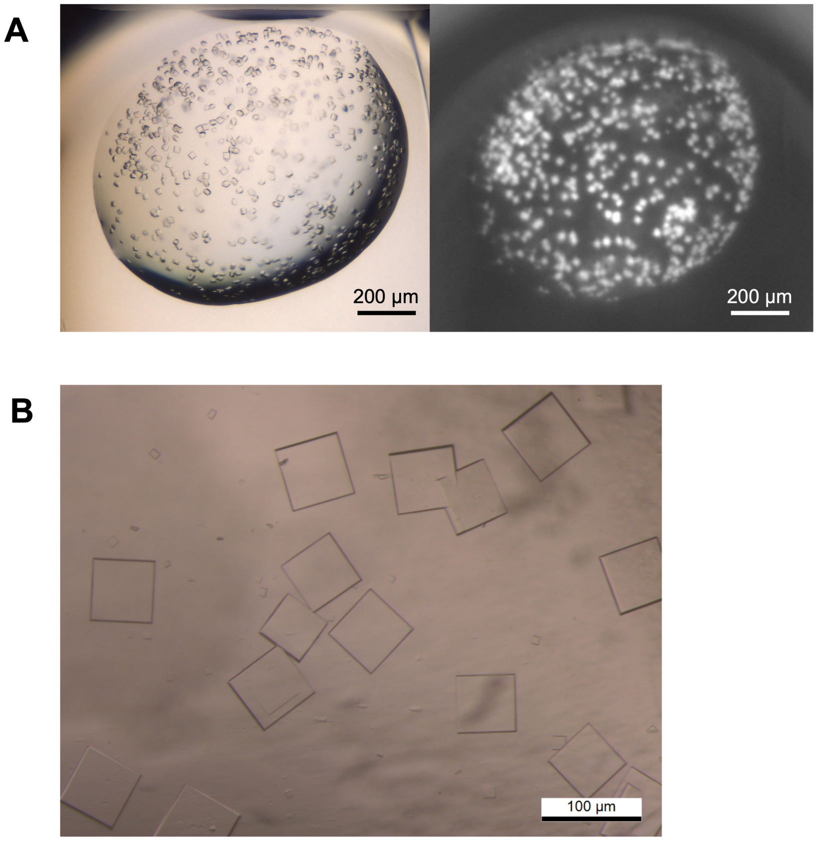


**Figure S5**. Erm38 protein crystals. (**A**) The initial hit from the JCSG+ crystallization kit. The protein crystals are confirmed upon UV exposure. (**B**) The optimal crystallization condition (1.0 M succinic acid pH7.0 and 10% glycerol) produced flat square crystals of ~50 µm in length.

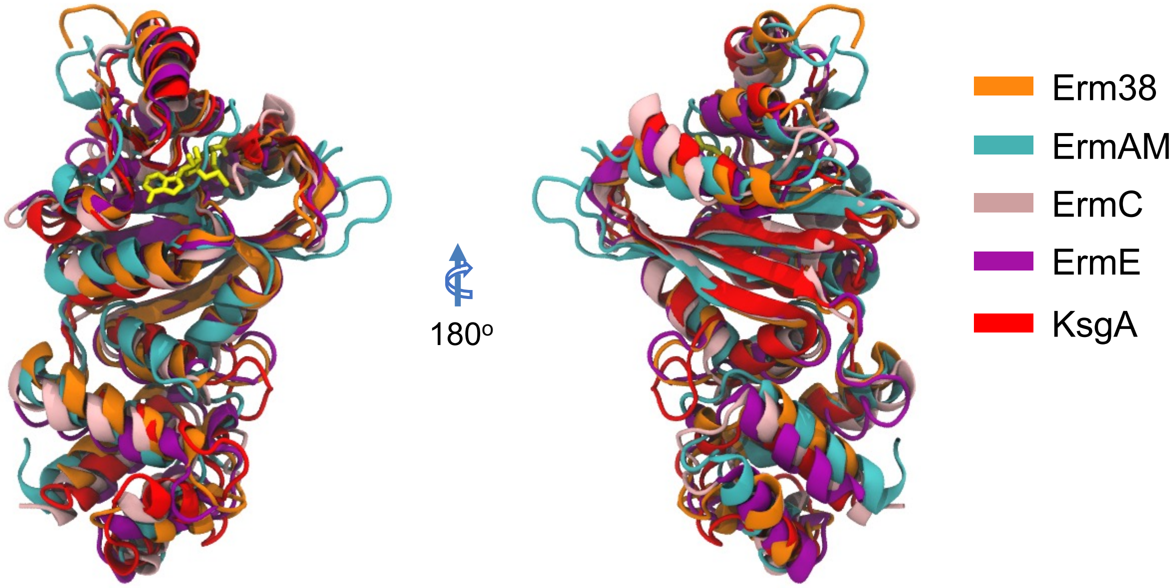


**Figure S6**: High structural similarity of Erm proteins and KsgA. Erm38 shown in orange ribbon is structurally similar to ErmAM (cyan), ErmC (pink), ErmE (purple) and KsgA (red). SAM molecule is shown in yellow stick. First 20 residues of KsgA that was not shown for clarity as this segment was unaligned with the other Erm structures.

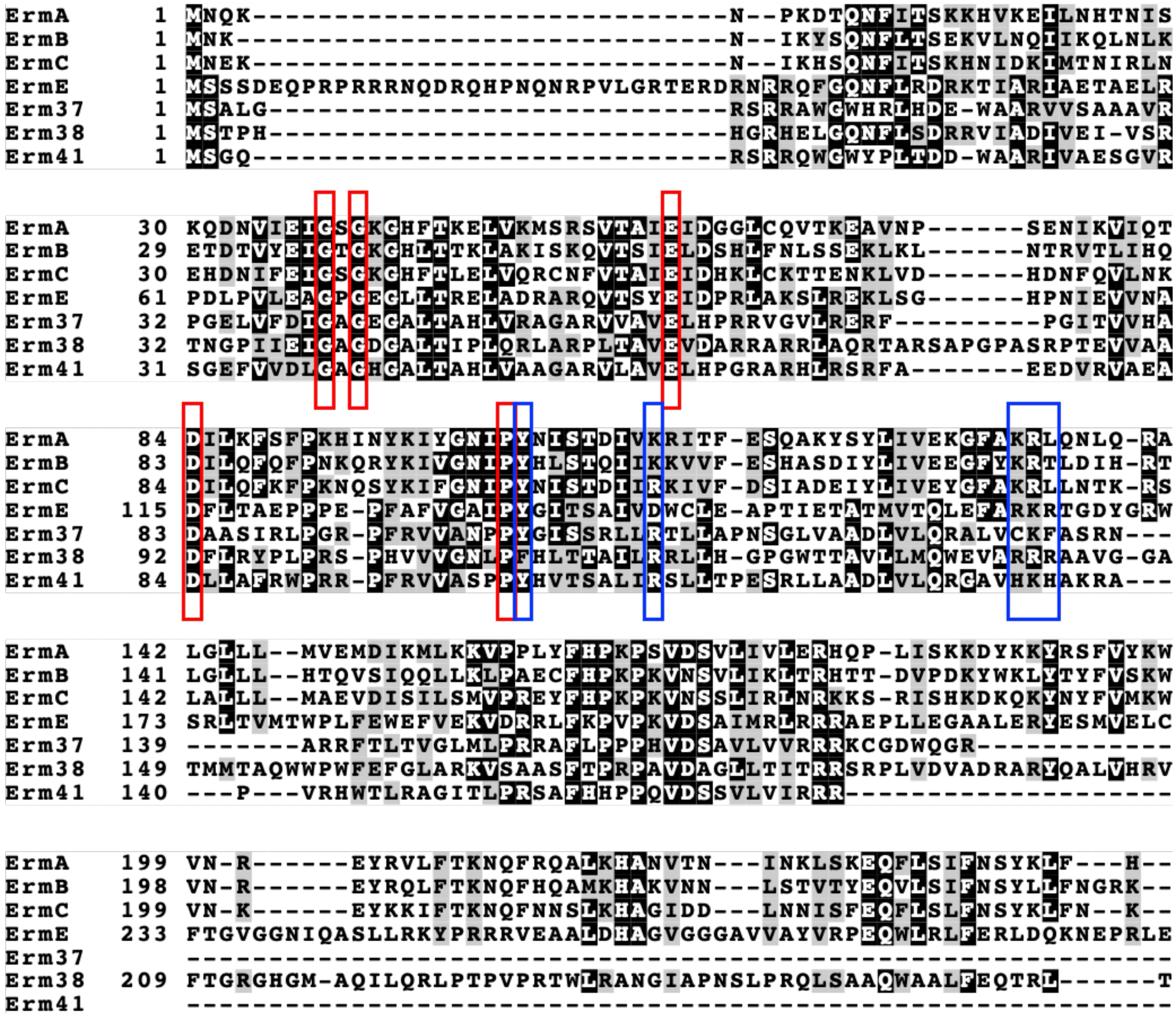


**Figure S7**: Sequence alignment of various Erm proteins. The red boxes highlight the conserved residues at the SAM binding pocket. The blue boxes highlight the residues that participate in binding to the RNA substrate. All of the boxed residues are highly conserved, except R142 of Erm38.

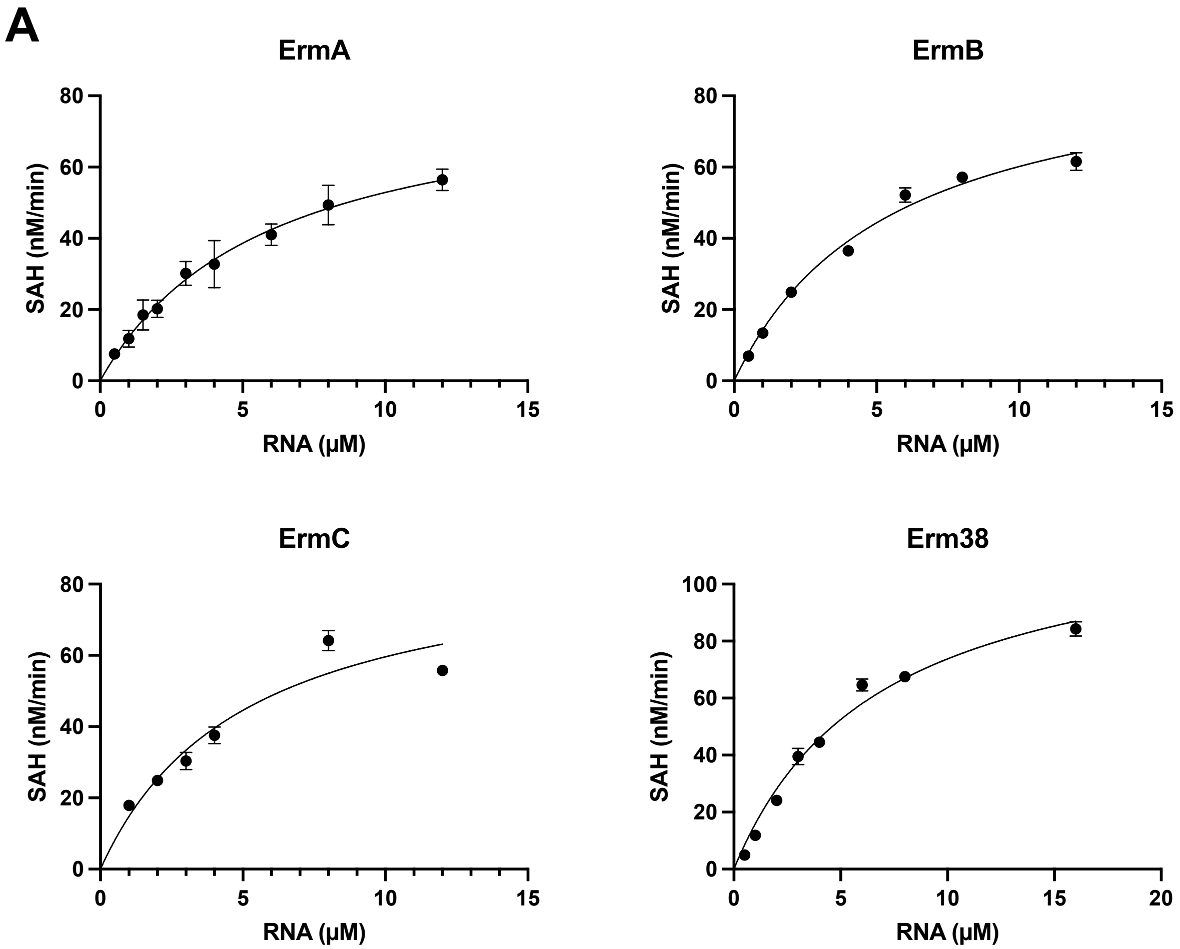


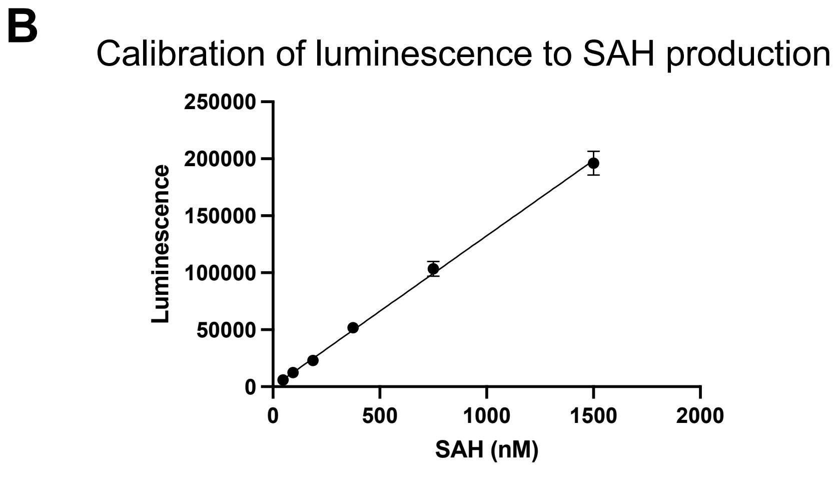


**Figure S8**: (A) Michaelis–Menten kinetics of various Erm protein binding to 32-mer RNA substrate. (B) Calibration curve to correlate the luminescence obtained on Promega MTase Glo kit to amount of SAM converted into SAH.

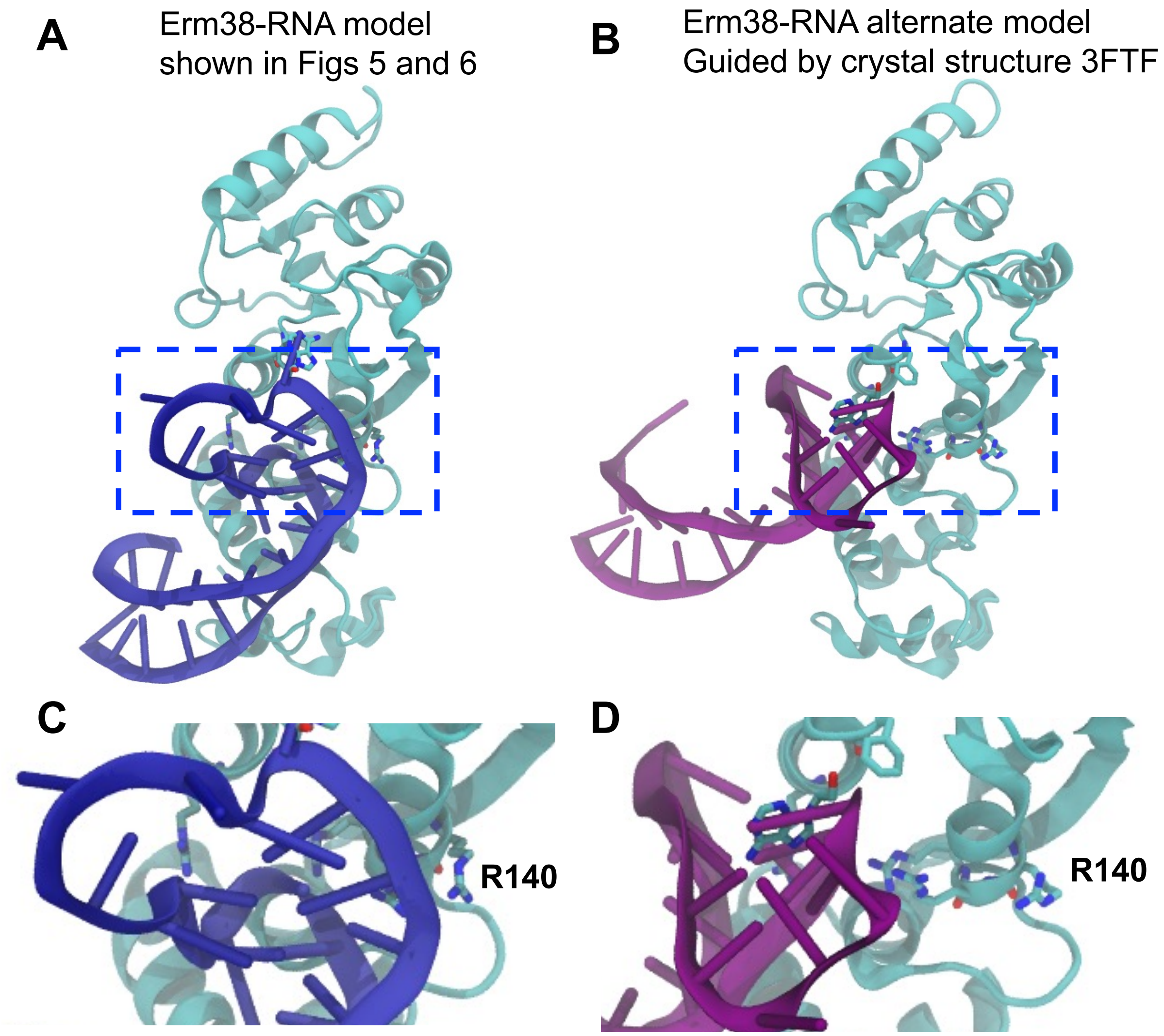


**Figure S9**: Comparison between two Erm38-RNA docked models. Model shown in panel A is better than model in panel B even though the modeling process is guided by the orientation of the KsgA-RNA complex (PDB accession number 3FTF). Panels C and D pinpoint the key difference between the two models. Panel D shows no interaction between the RNA and R140 of Erm38, an interaction that was shown to be crucial in our mutagenesis study.

```
Erm38     1 MSTPHHGRHELGQNFLSDRRVIADIVEIVSRTNGPIIEIGAGDGALTIPLQRLAR
Erm37     1 MSALGRSRRAWGWHRLHDEWAARVVSAAAVRPGELVFDIGAGEGALTAHLVRAGA
Erm41     1 MSG-QRSRRQWGWYPLTDDWAARIVAESGVRSGEFVVDLGAGHGALTAHLVAAGA

Erm38    56 PLTAVEVDARRARRLAQRTARSAPGPASRPTEVVAADFLRYPLPRSPHVVVGNLP
Erm37    56 RVVAVELHPRRVGVLRERFPG---------ITVVHADAASIRLPGRPFRVVANPP
Erm41    55 RVLAVELHPGRARHLRSRFAE-------EDVRVAEADLLAFRWPRRPFRVVASPP

Erm38   111 FHLTTAILRRLLHG-PGWTTAVLLMQWEVARRRAAVGGATMMTAQWWPWFEFGLA
Erm37   102 YGISSRLLRTLLAPNSGLVAADLVLQRALVCKFA-----SRN-ARRFT---LTVG
Erm41   103 YHVTSALIRSLLTPESRLLAADLVLQRGAVHKHA-----KRAPVRHWT---LRAG

Erm38   165 RKVSAASFTPRPAVDAGLLTITRRSRPL----
Erm37   148 LMLPRRAFLPPPHVDSAVLVVRRRKCGDWQGR
Erm41   150 ITLPRSAFHHPPQVDSSVLVIRRR--------
```

| | Erm38 | Erm37 | Erm41 |
|---|---|---|---|
| Erm38 | | 30 (41) | 30 (43) |
| Erm37 | 30 | | 57 (68) |
| Erm41 | 30 | 57 | |

**Figure S10**: Sequence alignment between the mycobacterial Erm proteins. Only the N-terminal of Erm38 (residues 1 to 192) is used for the alignment and calculation of sequence identity. The inset at the bottom right shows the sequence identity matrix of the Erm37, Erm41 with N-terminal of Erm38. Sequence similarity values are shown in brackets.

**Table S1**: primers used for the mutagenesis

| | Forward | Reverse |
|---|---|---|
| R31A | TCGATGATCGGACCGTTGGTAGCGGA AACGATTTCAACGATGTCC | GGACATCGTTGAAATCGTTTCCGCTACC AACGGTCCGATCATCGA |
| R130A | CTGGCTCGCCTGCTGCACGG | GTGGTCAGATGGAACGGCAGG |
| R151A | CCGTGCTGCGGTTGGCGG | CGAGCCGCCACTTCCCACTGC |
| R152A | CCGTGCTGCGGTTGGCGG | GCACGCGCCACTTCCCACTG |
| R153A | CGCTGCTGCGGTTGGCGG | CGACGCGCCACTTCCCACTGC |
| K177A | TTCTGCGGCGAGCTTCACCCC | ACTGCACGCGCCAAACCAAATTCAAAC |

Note that E61K was chemically synthesized by Bio Basic Inc.
